# Notes and News

**Published:** 1893-07-01

**Authors:** 


### Notes and News.

Collected and Communicated.

On July 17th the new laboratories and dental school buildings at Guy's Hospital are to be opened by the Right Hon. Sir John Lubbock.

Halifax is one of those towns so well provided with fever accommodation that the large number of small-pox cases which have had to be provided for over a year have not taxed the resources too greatly.. It is now deemed expedient to remove the hospital to another site, as the present one is not suitably placed.

At Leicester a great deal of friction is existent between the Municipal Council and the Fever Hospital. An inquiry originally was started into the cause of an outbreak of small-pox in the Fever Hospital, but this has spread itself into a hot discussion as to the merits and demerits of vaccination.

The "Liberator" Relief Fund is progressing, but the £100,000 required to rescue the many sufferers from poverty is a large sum to collect, and consequently it is desirable to enlist every possible aid in the cause. The class affected by this disastrous failure of a building society is one upon which the loss of money falls especially hard. In many cases the victims were entirely dependent upon the proceeds of their investment in the society, and to these the failure meant entire ruin. Possibly such persons are not the worst off, as friends are often found where distress is so apparent, and these claims would be the first to receive the attention of the relief fund. But to those who by self-denial and prudence have gradually accumulated the saving of years, in the hope of a provision when working days are over, or in the case of sickness, the blow was especially bitter. The incentive to hard work and self-denial must have departed from many, and the loss of courage and heart doubtless brought not a few victims to an untimely grave. If we can help the destitute and rekindle hope in the breasts of all the losers, we shall do much to restore the belief in the wisdom of saving for a rainy day so rudely shaken by the "Liberator" failure.

A Special meeting was held at St. George's Hospital on the 17th ult., under the presidency of the Earl of Cork and Orrery, to consider the desirability of erecting some fitting memorial to John Hunter, the centenary of whose death occurs this year. It was decided that this should take the form of a statue, to be placed where it might remind all who entered the hospital of his connection with it.

Messrs. Cubitt and Co., whose premises were recently injured by fire, write to Mr. Thies, the Secretary of the Royal Free Hospital, as follows: "Dear Sir,—We shall feel obliged if you will convey to the members of the staff of the Royal Free Hospital, including the lady students, and if you will also accept yourself our sincere thanks for the valuable service rendered by all on the occasion of the fire at our premises on Saturday last."

The Poplar Hospital for Accidents is to be closed in July. The management are negotiating with the London Hospital as to taking some of their cases. It is hoped that the new hospital will be ready in September. Fifteen thousand pounds has been raised towards the building fund, but £2,000 remains to be collected. The hospital possesses a most energetic chairman in the Hon. Sydney Holland, who spares no effort to further the interests of the institution.

The new chapel at the Cancer Hospital, Brompton, was opened recently by the Bishop of London. The whole of the £4,000 required for building the chapel was collected in four months, a fact which speaks well for the enthusiasm of those who made the appeal. The edifice is of red brick, having a curved roof of oak, and stained glass windows. It will seat about one hundred persons. The chapel was furnished by Sir Massey Lopes, who undertook this portion of the scheme, providing the funds for building were forthcoming. The opening ceremony commenced with Divine service. Later on a garden party was held in the hospital grounds.

To no one's particular loss, the once popular half-hour reading societies, almost exclusively patronised by young ladies, have mostly died out. A useful relic, however, lives and flourishes. This is the National Home Reading Union, whose head offices are at Surrey House, Victoria Embankment. This society is truly helpful to out of the way readers, for it draws up lists of carefully selected books. Members may also seek explanation when in the course of their reading they encounter difficulties. The object of the society is therefore to imperceptibly direct and cultivate the taste of young readers especially. The names of a few supporters of the society alone afford testimony to the good the society is likely to effect. In order to disseminate the objects of the society, summer assemblies are held annually. This year the meeting is to be at Ilkley, in Yorkshire, commencing on July 1st, and continuing for one week. Addresses will be read by the Master of Trinity College, Cambridge, Sir Robert Ball (the astronomer), Professor Michael Foster, Mrs. Henry Fawcett, and others. An illustrated programme, containing a time-table of lectures and meetings, list of lodgings and hotels, and full information on all subjects connected with the assembly, can now be had, price sixpence, from the Secretary.

